# RsaM: a unique dominant regulator of AHL quorum sensing in bacteria

**DOI:** 10.1099/mic.0.001417

**Published:** 2023-11-27

**Authors:** Vittorio Venturi, Mihael Špacapan, Nemanja Ristović, Cristina Bez

**Affiliations:** ^1^​ International Centre for Genetic Engineering and Biotechnology, Trieste, Italy

**Keywords:** quorum sensing, bacteria, RsaM, regulation, AHL, LuxI/R

## Abstract

Quorum sensing (QS) in proteobacteria is a mechanism to control gene expression orchestrated by the LuxI/LuxR protein family pair, which produces and responds to *N*-acyl homoserine lactone (AHL) diffusible signal molecules. QS is often regarded as a cell density response via the sensing of/response to the concentrations of AHLs, which are constantly basally produced by bacterial cells. The *luxI/R* systems, however, undergo supra-regulation in response to external stimuli and many regulators have been implicated in controlling QS in bacteria, although it remains unclear how most of these regulators and cues contribute to the QS response. One regulator, called RsaM, has been reported in a few proteobacterial species to have a stringent role in the control of AHL QS. RsaMs are small, in the range of 140–170 aa long, and are found in several genera, principally in *Burkholderia* and *Acinetobacter *. The gene encoding RsaM is always located as an independent transcriptional unit, situated adjacent to QS *luxI* and/or *luxR* loci. One of the most remarkable aspects of RsaM is its uniqueness; it does not fall into any of the known bacterial regulatory families and it possesses a distinct and novel fold that does not exhibit binding affinity for nucleic acids or AHLs. RsaM stands out as a distinctive regulator in bacteria, as it is likely to have an important ecological role, as well as unravelling a novel way of gene regulation in bacteria.

## Introduction

The current model of quorum sensing (QS) in bacteria relies on basal constitutive production of cell–cell signalling molecules leading to a bacterial population switch that synchronizes gene expression to coordinate social group behaviours [1, 2]. This model follows pioneering studies of the cell–cell signalling system of the marine bacterium *Aliivibrio fischeri* [3, 4]. This QS system depends on a diffusible *N*-acyl homoserine lactone (AHL) signal that accumulates when *A. fischeri* grows in the light organ of its symbiotic host squid *Euprymna scolopes,* resulting in the production of bioluminescence at high cell densities. The AHL signal is produced by the LuxI protein, which then binds to the LuxR regulator, which activates transcription of the *lux* operon, leading to bioluminescence. AHL QS is currently the most common cell–cell signalling system in proteobacteria, with many LuxI/R family systems identified, regulating many different social bacterial community phenotypes, such as biofilm, motility, production of secondary metabolites and virulence-associated factors [3, 5]. Although AHL QS is most commonly interpreted as a population density behaviour, these circuits are much more complex, being influenced by other physical, chemical and biological parameters of the environment, especially in bacteria inhabiting highly dynamic multi-species communities [6]. Consequently, the previously hypothesized population switch induced by AHL QS, as first observed in *A. fischeri *, is currently being questioned in various proteobacterial QS systems. In fact, supra-regulation has been reported for several AHL QS systems [7–10]. For example, the two extensively studied hierarchically organized systems of *Pseudomonas aeruginosa* are part of a complex regulatory network involving many other regulators (e.g. RpoS, RpoN, RsaL, PqsE) that affect their expression [10–13]. The QS systems of other bacteria, such as *A. harveyi*, *Ralstonia solanacearum* and *Rhizobium leguminosarum *, have also been evidenced to be under the control of other regulators, small RNAs and environmental cues [14–16]. Cyclic-di-guanosine monophosphate (cyclic-di-GMP) has been shown to play a role in triggering the QS response in *Sinorhizobium meliloti* and *P. aeruginosa* [17, 18]. Some AHL QS systems feature an intergenic element between the *luxI* and *luxR* genes that most often negatively and stringently affects the quantity of produced AHLs via regulation of the *luxI* family AHL synthase [19].

AHL QS studies in other pseudomonads have also evidenced extensive transcriptional and post-transcriptional regulation of *luxI/R* genes, but the stimuli and role of this regulation remain largely unclear [10, 12, 19, 20]. The large number of transcriptional and post-transcriptional regulators implicated in *luxI/R* regulation belong to known regulatory families, with one exception. A regulator of AHL QS that exerts a strong central regulatory role in a few proteobacterial species is called RsaM [19, 21–24]. RsaM orthologues are small proteins in the range of 140–170 aa long, implementing a key regulatory effect of AHL QS proteobacterial systems, and the encoding gene is always genetically organized as an independent transcriptional unit adjacent in tandem with *luxI* family and/or *luxR* AHL Q- related genes. The RsaM regulator that does not belong to any regulatory family and its regulatory mechanism is currently unknown and thus merits attention, as it could constitute a new regulatory family.

## Occurrence and genetic organization of *rsaM*/RsaM

**Fig. 1.**
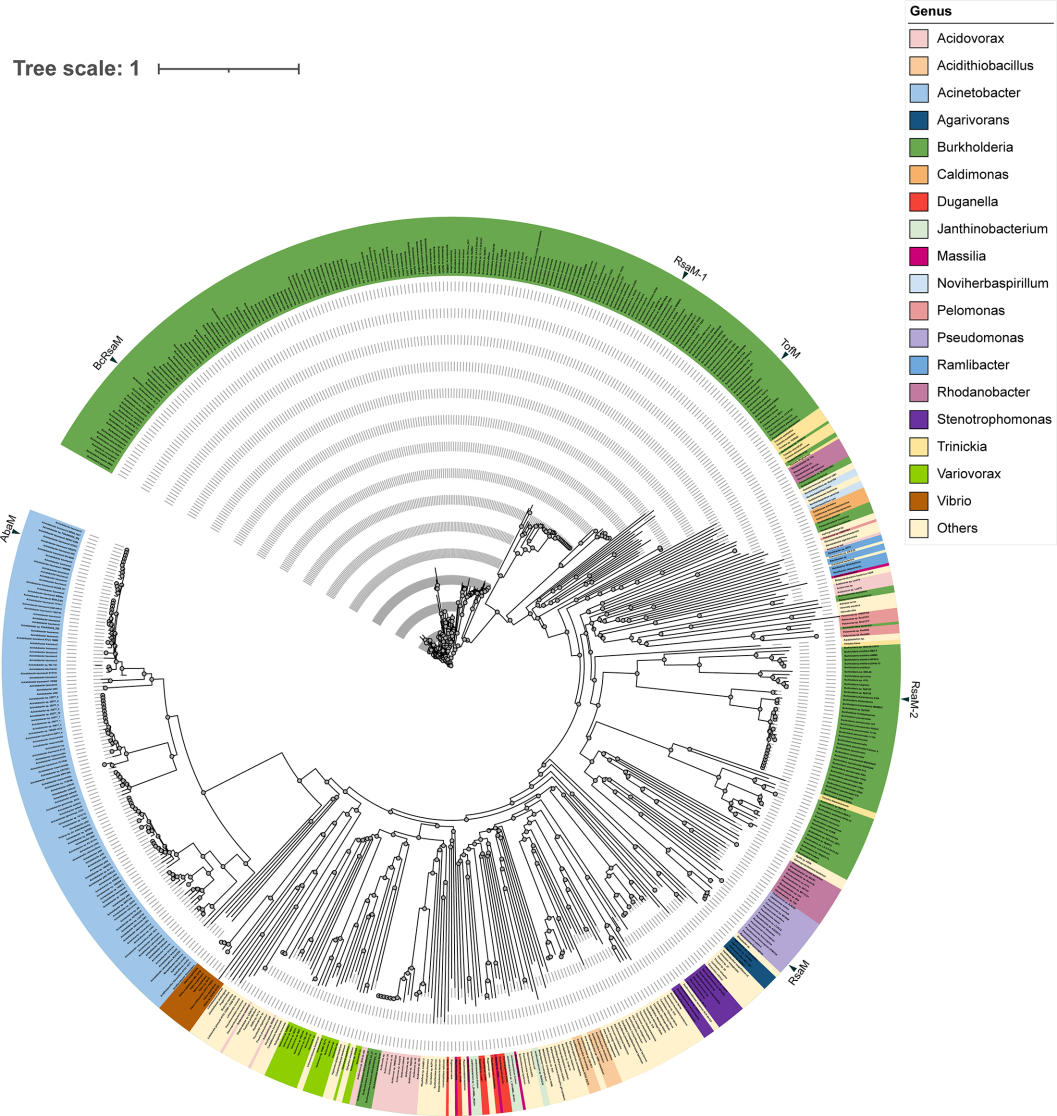
Taxonomic distribution and classification of RsaM in proteobacterial genomes. Genomes of proteobacteria from the IMG and NCBI databases were searched for the presence of *rsaM* genes (those that encode for proteins with domain PF16245). A tree of the RsaM representatives was made by aligning their protein sequences using clustal omega with standard settings [41], and then using FastTree with the alignment as input with standard settings [42]. The tree is annotated at genus level and the names of the best characterized RsaMs so far are indicated in the tree by an arrow in the external ring. Several hits belonging to bacterial genera that were detected as unique are grouped as ‘Others’. The tree is visualized and annotated using EMPress [43].

The *rsaM* gene has so far been reported in the genomes of β and γ proteobacteria that possess AHL QS systems. A bioinformatic analysis was performed here because it was of interest to determine the distribution of *rsaM* genes among microbial genomes deposited in the National Center for Biotechnology Information (NCBI) and the Joint Genome Institute Integrated Microbial Genomes (JGI IMG) databases[25, 26] following the identification of the RsaM protein domain pfam16245. pfam16245 is the only member of the superfamily cl24728 and there are no other proteins with similar domain architecture. RsaM orthologues are infrequent among bacterial genomes, thus far only being found in a few proteobacterial genera. RsaM was found in 62 proteobacterial genera, with different conservation and distribution; here are some examples organized in order of presence/abundance: *Burkholderia* (50.6 %), *Acinetobacter* (41.6 %), *Acidovorax* (0.74 %), *Pseudomonas* (0.47 %), *Variovorax* (0.40 %), *Acidithiobacillus* (0.34 %), *Trinickia* (0.34 %), *Rhodanobacter* (0.30 %), *Pelomonas* (0.21 %), *Vibrio* (0.15 %) and *Stenotrophomonas* (0.12 %). RsaM is most commonly found in *Burkholderia* and *Acinetobacter* spp., with the majority of *Burkholderia* genomes carrying more than one copy of an *rsaM* gene. However, considering that certain genera have a large number of genomes sequenced, while others have a limited representation in terms of sequenced genomes, it is premature to draw a conclusion that RsaM is sporadic in some of the genera in which it is present. The phylogenetic analysis shown in Fig. 1 indicates several clades based on the RsaM primary structure. The majority of RsaMs cluster according to the species taxonomy being conserved in the same genus (70–90 %), while RsaMs belonging to different genera showed a low primary structure relatedness; mostly around 25 %. In *Burkholderia *, multiple RsaMs are harboured by the same genome, but they do not cluster together, having low relatedness (15–22 %); these form two separate subgroups of RsaM (here called RsaMs1 and RsaMs2), which likely represent functionally related orthologues.

Importantly, *rsaM* genes were found to be always adjacent to the *luxI* and/or *luxR* loci, suggesting an evolutionary conserved primary function in QS regulation. Through our analysis, we identified eight different arrangements relative to the *luxI*/*R* QS loci (Table 1). As depicted and explained in Table 1, some topologies are much more frequent than others; in some cases *rsaM* is only associated with LuxR solos and in one case (topology type 8), very interestingly, it is encoded as part of a LuxR solo, resulting in a chimeric RsaM–LuxR solo protein, possibly indicating a functional role associated with QS LuxRs. In summary, RsaM genes have thus far been found predominantly in *Acinetobacter* and *Burkholderia* spp. genomes, consistently intimately associated with *luxI* and/or *luxR* genes, albeit in various topological arrangements.

**Table 1.** Schematic representation of the types of topological arrangements of the *rsaM* genes relative to the *luxI*/*R* genes. See footnote for details

Type*	Subtype*	Topology*	Taxonomic distribution*		
**1**		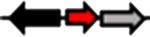	***Acidithiobacillus*** ***Budvicia*** ***Stenotrophomonas*** ***Trinickia***	*** Acinetobacter *** *** Methylobacter *** *** Pseudomonas *** *** Variovorax ***	***Burkholderia*** ***Leminorella*** ***Paraburkholderia*** ***Vibrio***
**2**		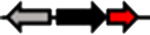	***Acidovorax***	***Rhodoferax***	***Pelomonas***
**3**	**a**	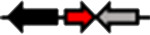	***Agarivorans***	***Aliagarivorans***	***Hydrogenophaga***
	**b**	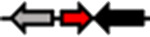	***Caldimonas***		
**4**	**a**	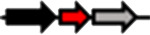	***Acidovorax*** ***Hydrogenophaga*** ***Thauera***	***Delphtia*** ***Paucibacter*** ***Variovorax***	***Candidimonas*** ***Diaphorobacter*** ***Schlegelella***
	**b**	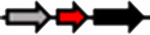	***Steroidobacter***		
	**c**	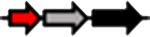	***Noviherbaspirillum***		
**5**	**a**	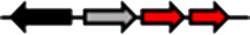	***Duganella***	***Janthinobacterium***	***Massilia***
	**b**	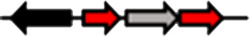	***Duganella***	***Janthinobacterium***	
**6**	**a**	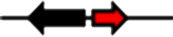	***Albitalea*** ***Thauera***	***Kinneretia*** ***Xenophilus***	***Pelomonas***
	**b**	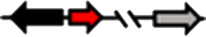	***Caballeronia***	***Methylobacter***	
**7**	**a**	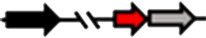	***Burkholderia***		
	**b**	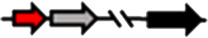	***Burkholderia***	***Chitiniphilus***	
**8**		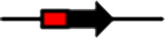	***Massilia***		

*Red arrows indicate *rsaM* genes (pfam16245), black arrows indicate *luxR* genes (pfam03472), grey arrows indicate *luxI* genes (pfam00765). In the vast majority of bacterial genomes, *rsaM* is present as an intergenic, independent transcriptional unit between *luxI* and *luxR* and in tandem with *luxI* (type 1). This is the only topology type found among bacteria belonging to the genera *Acinetobacter* and *Pseudomonas*. In the genus *Burkholderia*, the *rsaM* topology reflects the clustering of RsaM proteins into two distinct subgroups based on their primary structure; RsaMs1 are encoded by the *rsaM* genes in type 1 arrangements (Fig. 1). The wide distribution of this topology and its presence in bacteria with phylogenetically very distant RsaMs suggests that this is the most conserved arrangement among the QS systems possessing *rsaMs*. Importantly, in the table only some bacterial genera are reported as having this type of arrangement, but many others exist. On the other hand, the *rsaM* genes in type 2 arrangements are not intergenic, but rather in tandem with an upstream *luxR* gene that is adjacent to a *luxI* gene oriented in the opposite direction. This topology is found exclusively among bacteria belonging to the genera *Acidovorax* and *Rhodoferax*, with the only exception being a strain belonging to the genus *Pelomonas*. The *rsaM* genes with type 3 topological organization are intergenic, but with both *luxI* and *luxR* oriented in the opposite direction relative to the *rsaM*. Depending on whether the *rsaM* gene is divergent relative to a *luxR* or *luxI* gene, these topologies were further grouped into subtype 3a (*Agarivorans*, *Aliagarivorans*, *Hydrogenophaga*) or 3b (*Caldimonas*). The *rsaM* genes belonging to the type 4 topology are oriented in the same direction as *luxI* and *luxR* and three subtypes could be identified; most are grouped into subtype 4a, with *luxR* upstream and *luxI* downstream of the *rsaM*. While multiple *rsaM* genes harboured in the same genome usually belong to separate QS circuits and different gene topologies (*Burkholderia*, *Methylobacter*, etc.), a number of strains belonging to the genera *Duganella*, *Massilia*, *Janthinobacterium* and *Rugamonas* possess two *rsaM* genes that appear to be part of the same QS system (type 5) and are in tandem with a *luxI*, and in the opposite direction relative to a *luxR* gene. In addition, two different subtypes were detected for this type of arrangement, characterized by the presence of two copies of *rsaMs* that, surprisingly, shared a very low level of homology. The *rsaM* genes with type 6 genetic organization are adjacent to *luxR*, oriented in the opposite direction, with *luxI* being either absent from the genome or located at a distance. In subtype 6, *rsaMs* are adjacent to a *luxR* solo and are only present among bacteria within the genera *Pelomonas*, *Thauera*, *Kinneretia*, *Albitalea* and *Xenophilus*. The divergent *rsaM–luxR* gene pair is also present among a few bacterial species belonging to the genera *Methylobacter* and *Caballeronia* with an unusual QS gene topology in which the gene for a putative cognate *luxI* can be from 2 to 18.5 kb away. The *rsaM* genes in the type 7 arrangement are present in tandem with adjacent *luxI*, while a putative cognate *luxR* is located from 3 to 33 kb away from the pair, representing another atypical organization of QS genes, found within the genera *Chitiniphilus* and *Burkholderia*. RsaMs2s of the genus *Burkholderia* are usually encoded by the *rsaMs* with type 7 topology. In two bacterial species belonging to the genus *Massilia*, the DUF4902 (pfam16245) is found as part of a large LuxR-like protein comprising the autoinducer-binding domain in-between the RsaM domain at the N-terminal and the DNA-binding domain at the C-terminal (type 8). No genes belonging to the *luxI* family are present near the gene encoding the RsaM–LuxR chimeric protein, indicating that it most likely functions as a LuxR solo. This rare scenario suggests that RsaM could be linked with LuxRs via protein–protein interactions.

## RsaM is a regulator of AHL QS systems

RsaM has thus far been studied in a few proteobacteria where it plays a central regulatory role in AHL QS systems. It was first reported in the phytopathogen *Pseudomonas fuscovaginae *, which possesses two AHL QS systems, one of which is stringently negatively regulated by RsaM [24]. The *pfsI/R* AHL QS system is flanked by the *rsaM;* when *rsaM* is inactivated, there is a very strong increase in PfsI-synthesized AHLs, accompanied by a dramatic increase in *pfsI* transcription. There are two possible ORFs of *rsaM*, which are found in the same reading frame, one resulting in a 38 aa longer polypeptide in its *N*-terminus. The reported *rsaM* mutant contains the Tn*5*-inactivating insertion at the *N*-terminus of the longer ORF, indicating that RsaM is possibly translated as the larger ORF or alternatively the Tn*5* insertion results in deregulation of *rsaM*, as it could be inserted in the untranslated gene promoter region. Under laboratory conditions, *P. fuscovaginae* produces PfsI-AHL levels that are exceptionally low. This indicates that RsaM exerts stringent control, raising questions about the conditions under which QS becomes active. It is also possible that RsaM plays a role in allowing heterogeneity in the regulation, thus influencing the AHL response as explained below. Interestingly, *P. fuscovaginae* also harbours another AHL QS system called PfvI/R, which is stringently directly transcriptionally repressed by the DNA-binding repressor RsaL [24, 27, 28]. RsaM and RsaL proteins do not share any significant homology in primary and tertiary structure, indicating that they are not related. RsaM in *P. fuscovaginae* has also been associated with *in planta* pathogenicity, as demonstrated by the altered rice virulence observed in RsaM mutants [24].

RsaM has recently been reported in the opportunistic nosocomial pathogen *Acinetobacter baumannii *, which causes a wide range of infections in humans [23]. *A. baumannii* possesses the *abaI/R* AHL QS system and *rsaM*, which has been designated *abaM*, is located adjacent to *abaI* [23]. The *abaM* knockout mutant results in 100–800× more AHL production, indicating a very stringent and strong negative regulation. In addition, the AbaM mutant produced 3× more biofilm and is less virulent in a disease model system. This latter observation is an indication of the importance of stabilizing negatively regulatory pathways, as large increases in AHL production can lead to the emergence of less virulent mutants [19, 29]. Similarly to what was observed in *P. fuscovaginae *, AHL levels in wild-type *A. baumannii* are extremely low under laboratory conditions (<1 nM), again raising the question of when and under which conditions AHL QS is active, signifying that it might be switched off, with AbaM exercising a very tight control.

RsaM is widespread in *Burkholderia* spp.; the human pathogen *B. pseudomallei* and the closely related harmless soil saprophyte *B. thailandensis* both possess three AHL QS systems and two RsaM orthologues [22, 30, 31]. The three AHL QS systems in *B. thailandensis* are designated BtaI1/BtaR1, BtaI2/BtaR2 and BtaI3/BtaR3 and are organized hierarchically and integrated in a complex regulatory network [22]. System 1 and system 2 contain a *rsaM* homologue located in-between the *luxI/R* family genes designated *rsaM1* and *rsaM2*. RsaM1 has a significant negative impact on the transcription of *btaI1*, and RsaM2 on that of *btaI2*. Consequently, *rsaM1* and *rsaM2* mutants result in a considerable increase in the levels of AHL synthesized by BtaI1 and BtaI2, respectively. Interestingly, RsaM1 had a far greater effect on AHL concentrations than on *btaI1* transcription levels, indicating that RsaM1 could also be acting at the post-transcriptional level.

In the rice grain plant pathogen *B. glumae *, the TofI/R AHL QS system regulates the production of the phytotoxin toxoflavin, which is the major virulence factor [21, 32, 33]. Single-deletion mutants of *tofR*, *tofM* (*rsaM* orthologue) and *tofI* loci reduced the levels of biosynthesis of toxoflavin; a double *tofI/tofR* knockout mutant had residual low production of toxoflavin, whereas a triple *tofR/tofM/tofI* mutant displayed no measurable levels of toxoflavin [21]. TofM therefore acts as a positive regulator of toxoflavin production in the absence of TofR; this is in contrast to the inhibitory effects so far observed with the other RsaMs, as described above, although regulon studies in other bacteria have indicated that RsaM can also act as a positive regulator (see below).

## RsaM is a global regulator

Transcriptomic studies revealed that in *P. fuscovaginae* and *A. baumannii* RsaM also controls gene expression independently of its effect on QS [23, 34]. In *A. baumannii,* AbaM positively regulates 52 loci and negatively regulates 24; 21 of the upregulated loci are shared with the AbaI/R AHL QS system, whereas none of the negatively AbaM regulated targets are regulated by QS [23]. In *P. fuscovaginae *, RsaM regulates 466 genes, 56 % of which (260 genes) are positively regulated and 44 % (206 genes) are negatively regulated; 16 % of this RsaM regulon is also part of the AHL QS regulon, hence RsaM regulates a large number of loci independently of QS. The RsaM QS-independently regulated loci in *P. fuscovaginae* are involved in many important functions (including primary metabolism), such as carbon transport and metabolism, lipid transport and metabolism, amino acid transport, transcription and cell motility. In *A. baumannii,* AbaM QS-independently regulates loci involved in stress response, iron acquisition, several metabolic pathways and energy production, chaperones, protein folding and antibiotic resistance.

RsaM family proteins therefore behave as global regulators affecting the transcription of a large number of genes beyond the indirect regulation of AHL QS target loci. This indicates that RsaMs play an important role in programming gene expression in bacteria.

## RsaM possesses a novel fold and does not bind DNA or AHLS

RsaM currently shares no primary structure similarity to any structurally or biochemically characterized known proteins. The crystal structure of a *B. cepacia* RsaM has shown that it is a one-domain protein with the core consisting of a five-stranded anti-parallel β sheet that wraps around two α helices [35]. RsaM exists in a dimeric form and possesses a novel fold, characterized by a highly distinctive feature: a hydrophobic core cluster containing a conserved quartet of tryptophan residues (in some rare cases the quartet is not present). It does not contain any known DNA-binding motif, enzyme active site signatures or RNA-binding motifs. DNA-binding studies showed no binding to double-stranded DNA and experiments have also shown that it does not interact with AHLs [35]. Therefore, the mode of action is likely to involve other protein components of the transcription and/or translation machinery and protein–protein interaction studies will likely shed some light on the RsaM interactome and help reveal the mechanism by which it affects gene transcription.

## Hypotheses on the ecological role of RsaMs

In the two most studied examples discussed above, *rsaM* genome knockouts significantly increased the production of AHLs in *P. fuscovaginae* and *A. baumannii* [23, 24]. Both wild-type strains produce very low levels of AHLs in laboratory conditions and the QS systems can be considered inactive. Why would natural selection favour the emergence of such stringent repression? QS-regulated traits represent a considerable energy investment, therefore QS repression can be beneficial [19], particularly in environments where there is no selective pressure favouring the QS response.

Many QS systems can generate phenotypically heterogeneous populations, which can enable bet-hedging strategies [36]. When the environment is non-selective for QS-regulated traits, the repressed subpopulation will benefit by saving energy. If the environment changes and becomes selective for QS-regulated traits, the QS-active subpopulation will perform better, increasing fitness. The negative QS regulation by RsaMs could therefore play a role in phenotypic heterogeneity.

If a bacterial population can sense and predict the emergence of a selective pressure, it can also trigger the QS response according to that sensory input. In fact, many QS systems are logic AND gates, and therefore require additional triggers alongside AHLs [37]. This additional cue/condition can enable the bacterium to invest energy in a QS response only when needed; the additional conditions could either directly or indirectly lift RsaM-dependent repression and trigger the QS response. Because evolution usually occurs in smaller, gradual changes, it is very likely bacteria first evolved a bet hedging mechanism, and later refined it into a more complex response, gradually optimizing energy expenditure. In this case, RsaMs could be considered as sensors, which relieve QS repression after interacting with an environmental cue.

Finally, the repressive function of RsaMs could be present in bacteria because sometimes the environment warrants a blitz response, where many bacteria would suddenly and promptly begin manifesting a trait. In such a scenario, RsaM could function as an anti-activator, like those found in *P. aeruginosa* (i.e. QslA, QteE, QscR) [38, 39].

We assume that the QS system of *P. fuscovaginae* could be one of those that exhibits phenotypic heterogeneity, as recently discussed [40]. The idea is that RsaM functions as extreme negative feedback in conjunction to the positive feedback loop of the QS system. Such systems usually generate phenotypic heterogeneity, as was also shown and discussed recently [11]. When grown in the laboratory, in this case, the negative feedback loop is too strong, and hence no cells have an ‘active QS’ system. Most likely *in natura,* where the growth conditions differ dramatically, some of the cells manage to overcome the putative *rsaM* negative feedback and turn on due to an existing unknown stimulus/factor. Additionally, the system could still enable signal propagation, meaning that the putative signalling stimulus/factor is wired with AHL as an OR logic gate to the response machinery and not as an AND logic gate. If this is the case, ‘kick starting’ the culture with exogenous AHLs should produce such a phenotype. RsaM would therefore have a role in maintaining phenotypic heterogeneity. The hypothesis above can be tested by measuring the AHL QS dose–response in wild phenotype strains. If RsaMs generate phenotypic heterogeneity, we will observe the emergence of a stable subpopulation with an active QS. If RsaMs are sensors requiring an additional condition to be met, no response will be observed, and if RsaMs serve as anti-activators, the entire population will become QS active. Even if the different RsaMs are orthologous however, they could still have any of the functions above or be at different RsaM evolutionary stages. The latter mostly depends on the type of selective pressure that was exerted onto the common ancestor strain that carried the proto-version of RsaM. Further experimentation will therefore bring more clarity regarding the role of each of the specific RsaMs that have been discovered so far.

## Conclusions and unknowns

**Fig. 2.**
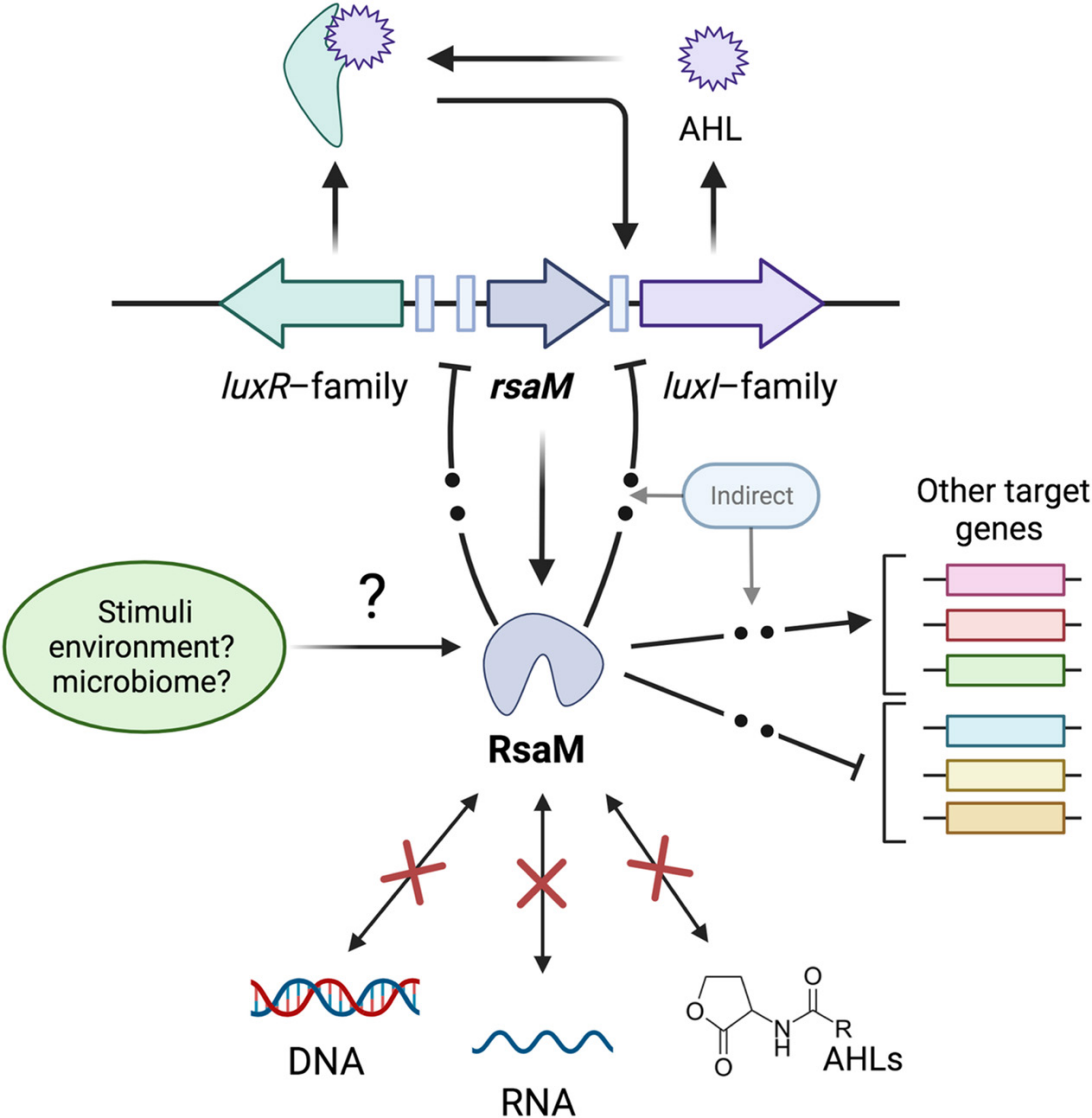
Diagrammatic representation of the mode of action of RsaM. Model of current knowledge on the RsaM regulator; see text for details. Dashed arrows represent a likely indirect regulatory mechanism.

The AHL QS response is energy expensive and is regulated to exploit environmental resources; the RsaM regulator stands out as a novel and unique player in ensuring adaptation of AHL QS to physical and biological cues. RsaM is always genetically linked to the AHL QS systems, most commonly as a unique transcriptional unit upstream in tandem with the *luxI* family synthase gene, while the protein has a unique fold and, despite playing a major role in transcriptional regulation, it does not bind DNA or RNA. The presence of *rsaM* immediately upstream of the *luxI* family gene could also encompass a *cis*-regulatory role in the transcriptional regulation of the *luxI* family AHL synthase. The lack of similarity to known proteins makes it difficult at this stage to speculate on its mechanism. RsaM is commonly found in *Burkholderia* and *Acinetobacter* and is also present in other proteobacterial species; the sequencing of more bacterial genomes will provide a clearer picture of its distribution, while advances in machine learning and experimentally determined structures will allow us to predict structural and hence functional similarities between RsaM proteins. RsaM behaves like a stringent QS central regulatory switch operating in the ecological sociomicrobiology context by possibly responding to environmental or microbiome cues and/or in a stochastic manner, allowing a bet-hedging strategy for QS. Several other types of negative regulators have also been reported that work using different mechanisms in concert with positive feedback loops in order to govern QS activation (see [11] and references therein). RsaMs are more global transcriptional regulators of a large number of functionally different genes beyond the indirect regulation of AHL QS target loci. Fig. 2 summarizes current knowledge of RsaM; this one-of-a-kind regulator calls for more research for its mode of action, as it will most probably result in a novel mechanism of regulation of gene expression in bacteria.

## References

[1] Bassler BL (2002). Small talk. Cell-to-cell communication in bacteria. Cell.

[2] Fuqua WC, Winans SC (1994). A LuxR-LuxI type regulatory system activates *Agrobacterium* Ti plasmid conjugal transfer in the presence of a plant tumor metabolite. J Bacteriol.

[3] Fuqua C, Greenberg EP (2002). Listening in on bacteria: acyl-homoserine lactone signalling. Nat Rev Mol Cell Biol.

[4] Nealson KH, Platt T, Hastings JW (1970). Cellular control of the synthesis and activity of the bacterial luminescent system. J Bacteriol.

[5] Schuster M, Sexton DJ, Diggle SP, Greenberg EP (2013). Acyl-homoserine lactone quorum sensing: from evolution to application. Annu Rev Microbiol.

[6] Mukherjee S, Bassler BL (2019). Bacterial quorum sensing in complex and dynamically changing environments. Nat Rev Microbiol.

[7] Frederix M, Downie AJ (2011). Quorum sensing: regulating the regulators. Adv Microb Physiol.

[8] Platt TG, Fuqua C (2010). What’s in a name? The semantics of quorum sensing. Trends Microbiol.

[9] Schuster M, Greenberg EP (2007). Early activation of quorum sensing in *Pseudomonas aeruginosa* reveals the architecture of a complex regulon. BMC Genomics.

[10] Venturi V (2006). Regulation of quorum sensing in *Pseudomonas*. FEMS Microbiol Rev.

[11] Mellini M, Letizia M, Caruso L, Guiducci A, Meneghini C (2023). RsaL-driven negative regulation promotes heterogeneity in *Pseudomonas aeruginosa* quorum sensing. mBio.

[12] Schuster M, Greenberg EP (2006). A network of networks: quorum-sensing gene regulation in *Pseudomonas aeruginosa*. Int J Med Microbiol.

[13] Williams P, Cámara M (2009). Quorum sensing and environmental adaptation in *Pseudomonas aeruginosa*: a tale of regulatory networks and multifunctional signal molecules. Curr Opin Microbiol.

[14] Baltenneck J, Reverchon S, Hommais F (2021). *Quorum sensing* regulation in phytopathogenic bacteria. Microorganisms.

[15] Calatrava-Morales N, McIntosh M, Soto MJ (2018). Regulation mediated by N-acyl homoserine lactone quorum sensing signals in the rhizobium-legume symbiosis. Genes.

[16] Ng WL, Bassler BL (2009). Bacterial quorum-sensing network architectures. Annu Rev Genet.

[17] Bettenworth V, van Vliet S, Turkowyd B, Bamberger A, Wendt H (2022). Frequency modulation of a bacterial quorum sensing response. Nat Commun.

[18] Schäper S, Krol E, Skotnicka D, Kaever V, Hilker R (2016). Cyclic Di-GMP regulates multiple cellular functions in the symbiotic alphaproteobacterium *Sinorhizobium meliloti*. J Bacteriol.

[19] Venturi V, Rampioni G, Pongor S, Leoni L (2011). The virtue of temperance: built-in negative regulators of quorum sensing in *Pseudomonas*. Mol Microbiol.

[20] Juhas M, Eberl L, Tümmler B (2005). Quorum sensing: the power of cooperation in the world of *Pseudomonas*. Environ Microbiol.

[21] Chen R, Barphagha IK, Karki HS, Ham JH, Arnold D (2012). Dissection of quorum-sensing genes in *Burkholderia glumae* reveals non-canonical regulation and the new regulatory gene tofM for toxoflavin production. PLoS One.

[22] Le Guillouzer S, Groleau M-C, Déziel E (2018). Two *rsaM* homologues encode central regulatory elements modulating quorum sensing in *Burkholderia thailandensis*. J Bacteriol.

[23] López-Martín M, Dubern J-F, Alexander MR, Williams P (2021). AbaM regulates quorum sensing, biofilm formation, and virulence in *Acinetobacter baumannii*. J Bacteriol.

[24] Mattiuzzo M, Bertani I, Ferluga S, Cabrio L, Bigirimana J (2011). The plant pathogen *Pseudomonas fuscovaginae* contains two conserved quorum sensing systems involved in virulence and negatively regulated by RsaL and the novel regulator RsaM. Environ Microbiol.

[25] Nordberg H, Cantor M, Dusheyko S, Hua S, Poliakov A (2014). The genome portal of the Department of Energy Joint Genome Institute: 2014 updates. Nucleic Acids Res.

[26] Chen I-M, Chu K, Palaniappan K, Ratner A, Huang J (2023). The IMG/M data management and analysis system v.7: content updates and new features.

[27] Rampioni G, Bertani I, Zennaro E, Polticelli F, Venturi V (2006). The quorum-sensing negative regulator RsaL of *Pseudomonas aeruginosa* binds to the lasI promoter. J Bacteriol.

[28] Rampioni G, Polticelli F, Bertani I, Righetti K, Venturi V (2007). The *Pseudomonas* quorum-sensing regulator RsaL belongs to the tetrahelical superclass of H-T-H proteins. J Bacteriol.

[29] Bondí R, Longo F, Messina M, D’Angelo F, Visca P (2017). The multi-output incoherent feedforward loop constituted by the transcriptional regulators LasR and RsaL confers robustness to a subset of quorum sensing genes in *Pseudomonas aeruginosa*. Mol Biosyst.

[30] Chandler JR, Duerkop BA, Hinz A, West TE, Herman JP (2009). Mutational analysis of *Burkholderia thailandensis* quorum sensing and self-aggregation. J Bacteriol.

[31] Choudhary KS, Hudaiberdiev S, Gelencsér Z, Gonçalves Coutinho B, Venturi V (2013). The organization of the quorum sensing luxI/R family genes in *Burkholderia*. Int J Mol Sci.

[32] Kim J, Kang Y, Choi O, Jeong Y, Jeong J-E (2007). Regulation of polar flagellum genes is mediated by quorum sensing and FlhDC in *Burkholderia glumae*. Mol Microbiol.

[33] Kim J, Kim J-G, Kang Y, Jang JY, Jog GJ (2004). Quorum sensing and the LysR-type transcriptional activator ToxR regulate toxoflavin biosynthesis and transport in *Burkholderia glumae*. Mol Microbiol.

[34] Uzelac G, Patel HK, Devescovi G, Licastro D, Venturi V (2017). Quorum sensing and RsaM regulons of the rice pathogen *Pseudomonas fuscovaginae.*. Microbiology.

[35] Michalska K, Chhor G, Clancy S, Jedrzejczak R, Babnigg G (2014). RsaM: a transcriptional regulator of *Burkholderia* spp. with novel fold. FEBS J.

[36] Bettenworth V, Steinfeld B, Duin H, Petersen K, Streit WR (2019). Phenotypic heterogeneity in bacterial quorum sensing systems. J Mol Biol.

[37] Veening JW, Smits WK, Kuipers OP (2008). Bistability, epigenetics, and bet-hedging in bacteria. Annu Rev Microbiol.

[38] Asfahl KL, Schuster M (2017). Additive effects of quorum sensing anti-activators on *Pseudomonas aeruginosa* virulence traits and transcriptome. Front Microbiol.

[39] Smith P, Schuster M (2022). Antiactivators prevent self-sensing in *Pseudomonas aeruginosa* quorum sensing. Proc Natl Acad Sci U S A.

[40] Spacapan M, Bez C, Venturi V (2023). Quorum sensing going wild. iScience.

[41] Sievers F, Wilm A, Dineen D, Gibson TJ, Karplus K (2011). Fast, scalable generation of high-quality protein multiple sequence alignments using clustal omega. Mol Syst Biol.

[42] Price MN, Dehal PS, Arkin AP (2009). FastTree: computing large minimum evolution trees with profiles instead of a distance matrix. Mol Biol Evol.

[43] Cantrell K, Fedarko MW, Rahman G, McDonald D, Yang Y (2021). EMPress enables tree-guided, interactive, and exploratory analyses of multi-omic data sets. mSystems.

